# Sustainable wastewater treatment by banana peel/layered double hydroxide composite under ideal conditions using the Taguchi method

**DOI:** 10.1038/s41598-026-37321-4

**Published:** 2026-02-17

**Authors:** Hamdy F. M. Mohamed, Sarah H. M. Hafez, E. E. Abdel-Hady, M. O. Abdel-Hamed

**Affiliations:** Physics Department, Faculty of Science, Minia University, P.O. Box 61519, Minia, Egypt

**Keywords:** Layered double hydroxide, Banana peels, Crystal violet, Adsorption, Ni-Ca-Fe/LDH, Wastewater, Low-cost alternative adsorbents, Chemistry, Engineering, Environmental sciences, Materials science

## Abstract

The present study investigates the removal of the cationic dye crystal violet (CV) from aqueous solutions using low-cost adsorbents derived from agricultural waste. Banana peels (BP) were activated, and a Ni–Ca–Fe layered double hydroxide (LDH) as well as a BP/LDH composite were synthesized and applied as adsorbents. The Taguchi experimental design was employed to optimize the adsorption process parameters, including pH, adsorbent dose, contact time, and initial dye concentration. Among the studied factors, pH, adsorbent dose, and initial concentration exhibited the most significant influence on CV removal efficiency. Under optimal conditions (pH 9, adsorbent dosage 0.1 g/L, and contact time 120 min), removal efficiencies of 66.0, 82.4, and 95.2% were achieved for BP, LDH, and BP/LDH, respectively. Analysis of variance (ANOVA) revealed that pH was the most influential parameter for CV adsorption onto BP and BP/LDH, whereas contact time played the dominant role for LDH. Equilibrium data were well described by the Freundlich, Langmuir, and Temkin isotherm models, with maximum adsorption capacities of 39.16, 81.10, and 187.40 mg/g for BP, LDH, and BP/LDH, respectively. Kinetic studies showed that the adsorption process followed the pseudo-second-order model, indicating chemisorption as the dominant mechanism. Reusability tests demonstrated that the BP/LDH composite retained a high removal efficiency of 80.0% after four adsorption – desorption cycles. These findings confirm that the BP/LDH composite is an efficient, sustainable, and reusable adsorbent with strong potential for practical wastewater treatment applications.

## Introduction

The rapid expansion of industrial and agricultural activities has significantly increased environmental pollution, representing one of the major global challenges of recent decades^1^. Among various pollutants, synthetic dyes are particularly problematic due to their toxicity and persistence, which can severely disrupt aquatic ecosystems by reducing light penetration and impairing photosynthetic activity^1,2^. Crystal violet (CV), a cationic triphenylmethane dye, is widely used in the textile, paint, medical, and biotechnology industries. Its discharge into water bodies is a major concern, as CV is known to be mutagenic, teratogenic, and cytotoxic^2,3^.

Effective removal of dyes from wastewater is therefore essential. Conventional treatment methods such as membrane separation, coagulation–precipitation, and advanced oxidation processes have been applied, but many of these approaches are limited by high cost, operational complexity, or secondary pollution^4,5^. Among the available technologies, adsorption has emerged as a simple, efficient, and cost-effective method for dye removal^5,6^. Activated carbon is widely used as an adsorbent due to its high surface area and adsorption capacity. However, its production is expensive and often involves chemical activation with agents such as potassium hydroxide or sodium hydroxide, which can leach into treated water^6,7^. These limitations have motivated the search for alternative low-cost adsorbents. Recently, a variety ofs materials have been explored as substitutes for activated carbon, including metal–organic frameworks (MOFs), hydrogels, clays^7–9^, nanomaterials^10^, and layered double hydroxides (LDHs)^2,11^. LDHs, also known as hydrotalcite-like compounds, have attracted considerable attention due to their unique layered structure, high surface area, and ion-exchange capacity^11,12^. The general formula of LDHs is [M^2 +^_1−*x*_M^3 +^_*x*_(OH)_2_]^*x*+^[A^*n*−^_*x*/*n*_]^*x*−^· *m*H_2_O, where M^2+^, and M^3+^ are divalent and trivalent metal cations, respectively, and A^*n*−^ is the interlayer anion^12^. LDHs can efficiently remove cationic or anionic pollutants through ion exchange, and their adsorption efficiency can be further enhanced via surface modification or compositing with organic and inorganic materials^11,13,14^. However, the high cost of commercial LDH-based adsorbents limits their large-scale application^14,15^.

To overcome these limitations, biomass-based adsorbents have emerged as sustainable alternatives due to their abundance, low cost, and rich surface functional groups. Fruit peels, in particular, offer a promising feedstock for bioadsorbents owing to their large surface area, availability, and environmental compatibility^16–18^. Banana peels (BPs), which account for 30–40% of the fruit’s weight, are rich in cellulose, lignin, minerals, and surface functional groups (hydroxyl, carboxyl, and amide), making them excellent candidates for dye adsorption^16,19,20^. The use of BPs not only provides an eco-friendly solution for wastewater treatment but also contributes to waste valorization, addressing both environmental contamination and resource management issues^17,21^. Several studies have reported the use of low-cost biomass materials—including grapefruit peel^22^, rice husk^23^, ginger waste^24^, and banana peel^16,19,20,25^—for the removal of CV dye. Pretreatment methods, such as carbonization or chemical modification with acids or alkalis, have been applied to enhance adsorption performance^16,26^. Despite the promising results, research integrating biomass adsorbents with LDHs remains limited, particularly in exploring synergistic effects to improve dye removal efficiency and reusability^11,12^.

In this study, we prepared banana peel (BP) and a banana peel/layered double hydroxide composite (BP/LDH) and investigated their effectiveness in removing crystal violet dye from aqueous solutions. The adsorbents were characterized using X-ray diffraction (XRD), Fourier-transform infrared spectroscopy (FTIR), scanning electron microscopy (SEM), and energy-dispersive X-ray spectroscopy (EDX). Key operational parameters—including pH, temperature, contact time, and dye concentration—were systematically evaluated. Adsorption kinetics and thermodynamics were analyzed to elucidate the underlying mechanisms, and the reusability of the adsorbents was assessed. By combining a low-cost biomass adsorbent with LDH, this work provides a novel, sustainable, and efficient approach for the remediation of cationic dyes from wastewater, addressing both environmental and economic challenges^3,11,16,19^.

## Materials and methods

### Materials

Sodium hydroxide (NaOH, 98.5%) and hydrochloric acid (HCl, 36%) were purchased from Chemlab NV Co. (Egypt). Ferric nitrate nonahydrate (Fe(NO_3_)_3_·9H_2_O), calcium nitrate hexahydrate (Ca(NO_3_)_2_·6H_2_O), and nickel nitrate hexahydrate (Ni(NO_3_)_2_·6H_2_O) were supplied by Loba Chemie (India). All chemicals were of analytical grade and used as received without further purification. Distilled water was used for the preparation of all aqueous solutions.

### Preparation of activated banana peels (BP)

**Scheme 1:**
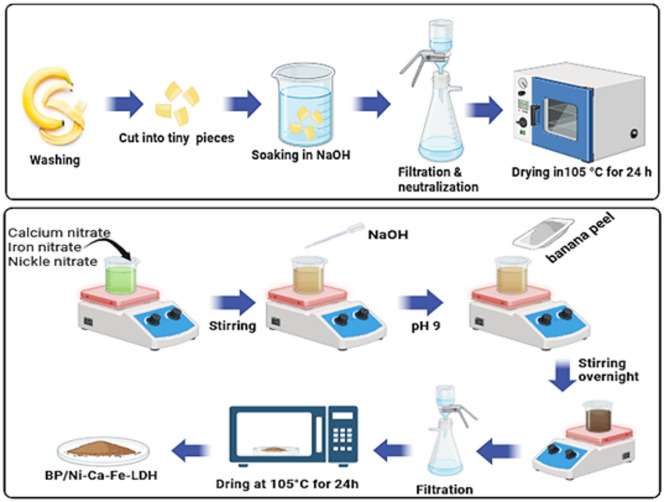
Preparation of adsorbent nanoparticles.

Raw banana peels (BP) were thoroughly washed with distilled water to remove adhering impurities, then cut into small pieces (0.5–1.0 cm). The cleaned peels were chemically activated by immersion in 1 M NaOH solution for 2 h to enhance surface functionality and adsorption efficiency. After activation, the samples were filtered and repeatedly washed with distilled water until neutral pH was attained. The treated material was then dried in an oven at 105 °C for 24 h, ground into a fine powder, and stored for subsequent use^27^. A schematic illustration of the adsorbent preparation procedure is shown in Scheme 1.

### Preparation of BP/LDH composite

The Ni–Ca–Fe layered double hydroxide (LDH) was synthesized using a co-precipitation method with a molar ratio of 4:2:1, respectively^28^. Appropriate amounts of nickel, calcium, and iron nitrate salts were dissolved in deionized water at room temperature (25 °C) under continuous stirring. A 1 M NaOH solution was gradually added to the mixed metal nitrate solution until complete precipitation occurred and the pH reached 9. Subsequently, 4 g of activated banana peel powder was added to the suspension, and the mixture was stirred overnight to obtain the BP/LDH composite with a BP: LDH weight ratio of 70:30. The resulting product was filtered, washed, and dried at 105 °C for 24 h^29^.

### Adsorbent characterization

To determine the nature of the produced solid particle, chemical and textural examination performances were applied. A monochromator beam (λ = 1.5406 Å-CuKα radiation) and 40 kV with a current of 40 mA were used in a PANalytical (Empyrean) X-ray diffractometer to obtain the XRD pattern (X-ray diffraction). At a rate of 2θ = 2°/min, the angle (2θ) was measured from 5 to 80°. With a spectral resolution of 4 cm^−1^, the FTIR spectrum was obtained and the molecular vibration in chemical bonds was analyzed using a Mattson 5000 FTIR spectrometer. The 400–4000 cm^−1^ wavenumber range was used for collecting the infrared spectra. EDX and SEM images were processed using a JSM-IT200 microscope (Tokyo, Japan) to examine the samples’ morphology at 20 keV and high vacuum. The measurements were carried out at Minia University’s Central Laboratory for Microanalysis and Nanotechnology, Egypt. The CV dye concentration in the samples was established using an ultraviolet and visible spectrophotometer (Unico instrument-UV2000-USA).

### Adsorption studies

#### Experimental procedures

Batch adsorption experiments were conducted to evaluate the removal of crystal violet (CV) dye from aqueous solution. In each experiment, 25 mL of CV solution with an initial concentration of 20 ppm was mixed with a known dose of adsorbent and stirred at 25 °C for 300 min. After adsorption, the solid adsorbents were separated by centrifugation. The residual CV concentration was determined using a UV–Vis spectrophotometer (Unico UV-2000, USA). The removal efficiency (R, %) and equilibrium adsorption capacity (q_e_, mg/g) were calculated using Eqs. (1) and (2), respectively^4,9^:

1 $$\:R\%\:=\:\frac{{C}_{i}-\:{C}_{e}}{{C}_{i}}\:\times\:100,$$

2 $$\:{\:\:\:q}_{e}=\frac{({C}_{i}-{C}_{e})}{m}\:\times\:V.$$

Where *C*_i_ and *C*_e_ (mg/L) are the initial and equilibrium dye concentrations, respectively, *V* (L) is the volume of the solution, and *m* (g) is the mass of the adsorbent.

#### Design of experiment using the Taguchi method

The Taguchi method, originally developed by Genichi Taguchi, was employed to optimize the adsorption conditions and evaluate the influence of operational parameters on CV dye removal^30^. Compared to conventional optimization techniques, the Taguchi approach provides an efficient statistical framework to identify significant factors with a reduced number of experiments^31^. Optimization of CV dye removal was performed using an L9 orthogonal array design (Table 1). The experimental results were analyzed by analysis of variance (ANOVA) based on signal-to-noise (S/N) ratios using Minitab software (version 21)^32^. The S/N ratio is a dimensionless index used to assess the deviation of the experimental response from the desired performance^33^. In this study, the “larger-the-better” criterion was selected, as maximizing dye removal efficiency was the primary objective. The corresponding S/N ratio was calculated using Eq. (3)^34^:

**Table 1:** Studied process parameters and their levels.

Parameter	Level		
	1	2	3
pH	3	7	9
Dose (g)	0.03	0.1	0.3
Temperature (°C)	25	35	55
Time (sec)	5	60	120

3 $$\:\frac{S}{N}=-10\mathrm{log}\left[\frac{1}{n}\sum\:_{i=1}^{n}\frac{1}{{{y}_{i}}^{2}}\right].$$

Where *n* is the number of experiments, and *y* is the response of the variables^35^. Four main parameters, adsorbent dosage, solution pH, temperature, and contact time, were investigated using the L9 orthogonal array, and a total of nine experimental runs were conducted^36^. The experimental conditions for CV dye removal using BP, LDH, and BP/LDH are summarized in Table 1.

## Results and discussion

### Characterization of the prepared solid adsorbents

#### SEM

**Fig. 1:**
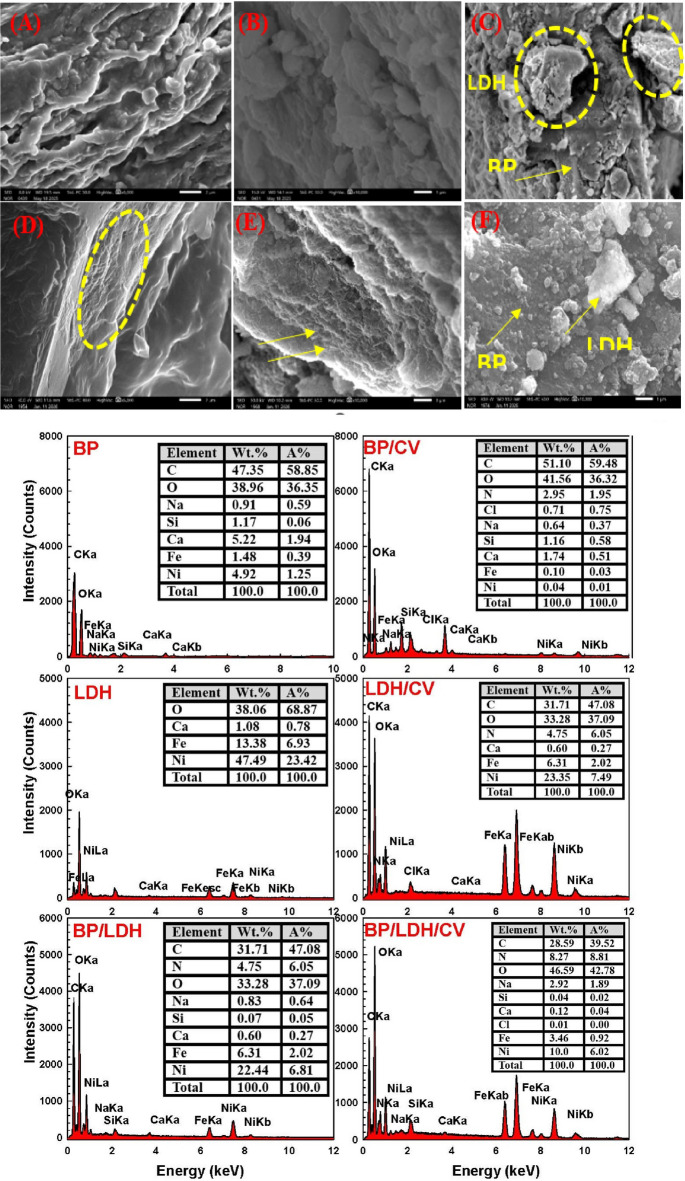
SEM images of (**A**, **D**) BP, (**B**, **E**) LDH, (**C**, **F**) BP/LDH before and after adsorption respectively, with EDX of the samples.

The SEM image of banana peel (BP) shown in Fig. 1A reveals a porous and irregular surface morphology. The SEM image of LDH (Fig. 1B) displays a rough surface with abundant pores and aggregates of plate-like nanoparticles stacked on top of each other. These structural features are expected to enhance dye adsorption by facilitating the diffusion of dye molecules into the internal structure of the material^37^. The SEM image of the BP/LDH nanocomposite (Fig. 1C) demonstrates a uniform distribution of LDH crystals on the BP surface, confirming the successful formation of the composite. Following CV adsorption, the SEM micrographs of (BP), (LDH), and the BP/LDH composite offer direct morphological proof of dye uptake on the adsorbent surfaces. Following the adsorption of CV dye, the BP surface seems less porous and more compact than it was before adsorption (Fig. 1D). The accumulation of CV molecules within the accessible pores and on the external surface is indicated by the partial filling or covering of several of the surface cavities and fibrous channels. Figures (1E) illustrated LDH after adsorption with a loss of distinctive layered plate-like shape. Partial particle aggregation is seen, and the margins of the LDH sheets appear coated with dye. On the other hand, the surface of the BP/LDH composite shows the most noticeable alterations upon adsorption (Fig. 1F). The majority of pores and surface imperfections are greatly reduced or blocked, and the composite becomes denser and more evenly covered.

The elemental composition of BP, LDH, and their composites before and after CV adsorption was examined using energy-dispersive X-ray spectroscopy (EDX), as shown in Fig. 1. Strong C and O signals dominate the EDX spectrum for BP, confirming its carbonaceous composition. The presence of carbon-containing functional groups on its surface also suggests that BP has a large carbon framework. According to the LDH sample, distinctive O peak is shown, in addition to metal signals like Ni, Ca, and Fe, indicating that the layered double hydroxide structure was successfully formed. EDX spectrum for BP/LDH composite displays the elemental composition of both components. The existence of elements indicates BP and LDH’s effective combination. Following CV adsorption, additional N-corresponding peaks can be noticed. The successful adsorption of CV onto the surface is directly demonstrated by this nitrogen signal, which comes from the CV dye molecules. Furthermore, the corresponding rise in C and O intensities following adsorption indicates that the dye molecules and the samples’ oxygenated functional groups interact strongly.

#### Morphological surface analysis

**Fig. 2:**
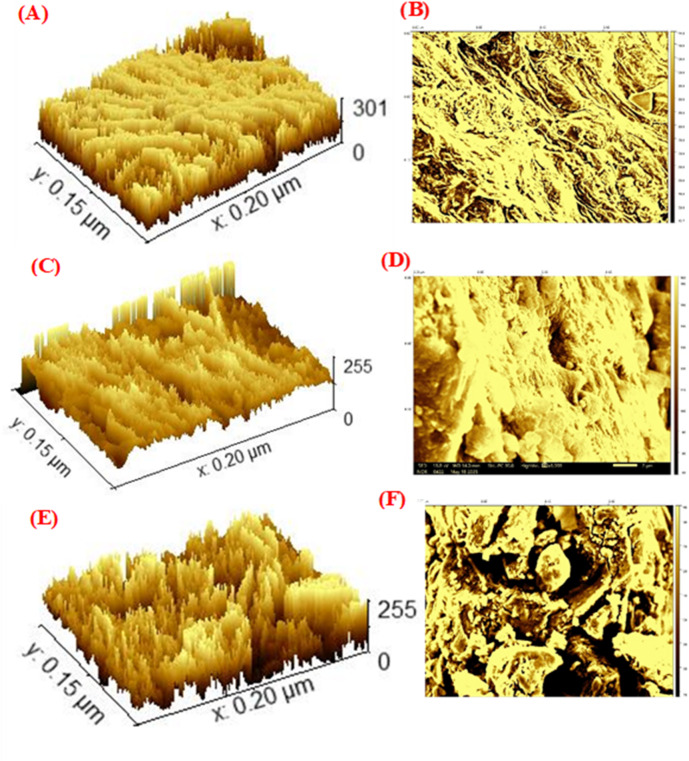
The 3D SEM micrographs and 3D topographical of the BP (**A**,**B**), LDH (**C**,**D**), and BP/LDH (**E**,**F**).

**Fig. 3:**
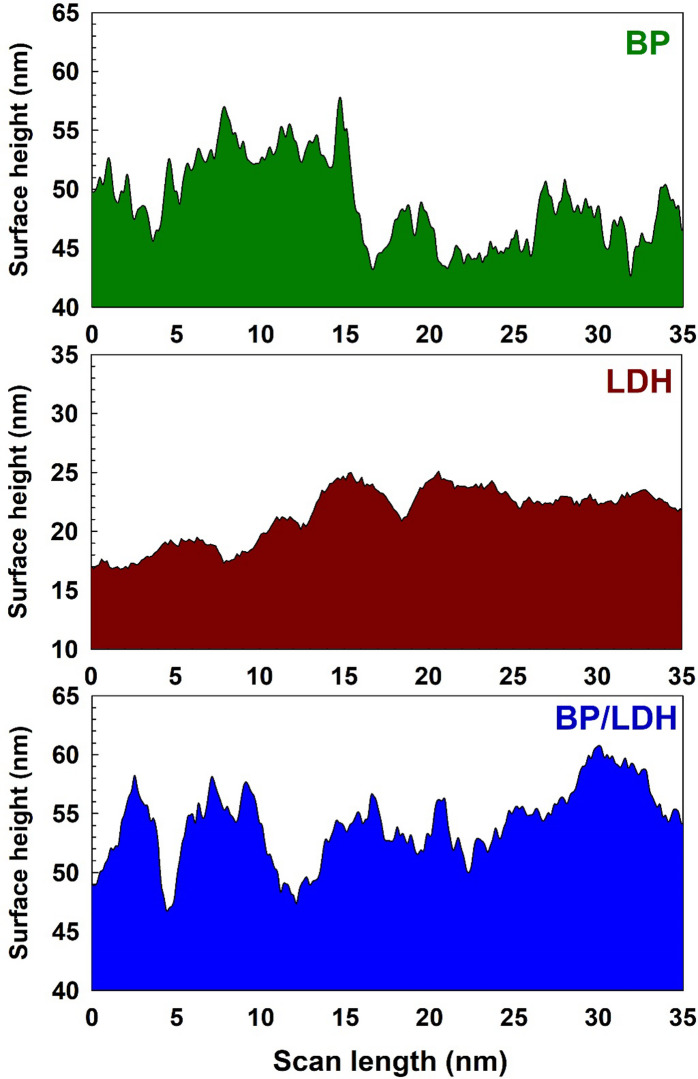
The profile’s heights of BP, LDH, and BP/LDH.

Since the surface morphology of LDH exhibits heterogeneities and irregularities that may influence its practical application, the SEM topographic images were further analyzed using Gwyddion 2.68 software^38^. The three-dimensional (3D) SEM micrographs and surface topography images of BP (A, B), LDH (C, D), and the BP/LDH composite (E, F) are presented in Fig. 2. The BP sample (A, B) exhibits a highly irregular and rough surface characterized by numerous peaks and valleys. These pronounced height fluctuations arise from the lignocellulosic structure of banana peels^39^. In contrast, the LDH sample (C, D) shows a distinctive plate-like and layered morphology typical of LDH materials, with brucite-like nanosheets stacked over one another, resulting in a relatively rough surface. The BP/LDH composite (E, F) combines the morphological features of both BP and LDH, forming a more complex and hierarchical structure due to the uniform deposition of LDH nanosheets onto the rough BP surface. This confirms strong interfacial interactions between BP functional groups and LDH layers. A schematic height distribution profile showing surface height versus lateral length is presented in Fig. 3. The BP/LDH composite exhibits the highest surface height variation (approximately 48–60 nm), indicating a rougher and more textured surface compared to the pristine samples. This increased roughness can be attributed to the successful integration of LDH layers onto the BP surface, creating a complex surface topology that is expected to enhance surface area and improve adsorption performance.

The roughness parameters of BP, LDH, and BP/LDH are summarized in Table 2, including root mean square roughness (RMS), average roughness (Ra), roughness skewness (Rsk), and roughness kurtosis (Rku). The kurtosis parameter (Rku) reflects the sharpness of the surface height distribution, where values below 3 indicate bumpy surfaces, values above 3 indicate spiky surfaces, and a value of 3 corresponds to a Gaussian distribution. All samples exhibit Rku values greater than 3, indicating spiked height distributions. The skewness parameter (Rsk) represents surface symmetry, with positive values indicating predominantly peaky surfaces and negative values indicating valley-dominated surfaces^4^. All samples show positive Rsk values, confirming peaky surface profiles. Notably, the BP/LDH composite displays the highest average roughness (7.639 μm) and the largest projected area (29.831 μm^2^), which reflect a significantly increased surface area. This enhancement suggests greater chemical reactivity and improved adsorption capability. Moreover, surface roughness may influence the chemical homogeneity of the composite, further affecting its functional performance.

**Table 2:** Textural characteristics of LDH, BP, and BP/LDH.

Parameters	BP	LDH	BP/LDH
Average roughness (Ra) (µm)	4.278	2.941	7.639
Root mean square roughness (RMS)	56.114	24.427	65.340
Roughness skewness (Rsk)	0.1838	0.0269	0.1869
Roughness kurtosis (Rku)	6.4721	3.5714	6.2065
Projected area (µm^2^)	27.298	28.603	29.831

#### FTIR

**Fig. 4:**
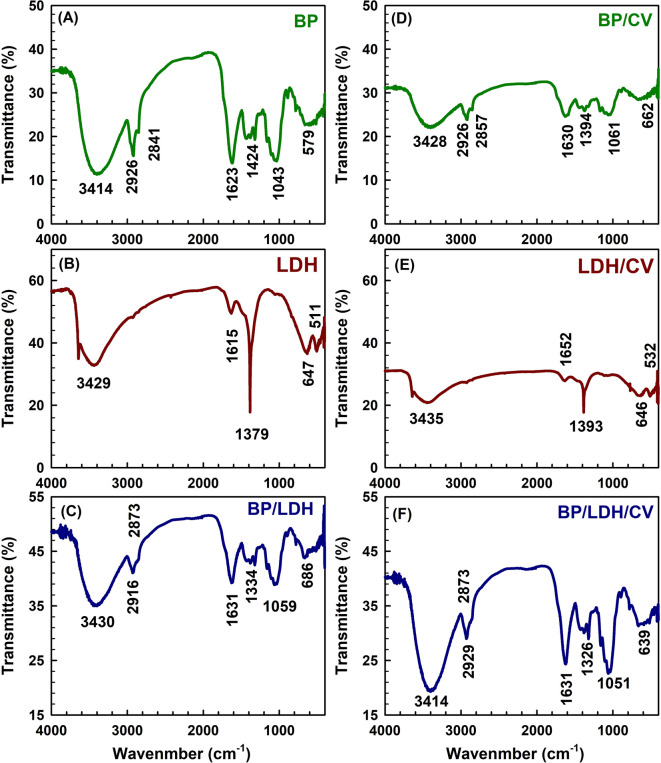
FTIR spectra of BP, LDH, and BP/LDH before and after CV adsorption.

The FTIR spectrum of BP, shown in Fig. 4A, exhibits several characteristic absorption bands corresponding to different functional groups. A strong and broad band centered at 3414 cm^−1^ is attributed to the O–H stretching vibrations of free hydroxyl groups in polymeric constituents such as cellulose and lignin^39^. The band at 2926 cm^−1^ corresponds to O–H stretching vibrations in amino acids, while the band at 2841 cm^−1^ is assigned to the C–H stretching of aliphatic hydrocarbons. The absorption band at approximately 1623 cm^−1^ is associated with C = O stretching vibrations of carboxylic groups (–COOH and –COOCH_3_), indicating the presence of carboxylic acids or their esters^40,41^. In addition, the band at 1043 cm^−1^ is attributed to C–H bending vibrations in crystalline cellulose and related polysaccharides such as hemicellulose and lignin, while the band at 1424 cm^−1^ is related to N–H bending vibrations. A weak band observed at 579 cm^−1^ further suggests the presence of amine groups.

The FTIR spectrum of the LDH sample (Fig. 4B) shows a broad band at 3429 cm^−1^ corresponding to the O–H stretching vibrations of both interlayer and adsorbed water molecules. The bands at 1615 and 1379 cm^−1^ are assigned to the asymmetric and symmetric stretching modes of nitrate ions (NO_3_−), respectively^9^. Furthermore, absorption bands below 1000 cm^−1^ are attributed to metal–oxygen (M–O), metal–oxygen–metal (M–O–M), and oxygen–metal–oxygen (O–M–O) vibrations, which are characteristic of the hydrotalcite-like LDH lattice structure, where M represents Ni, Ca, or Fe^25^.

**Scheme 2:**
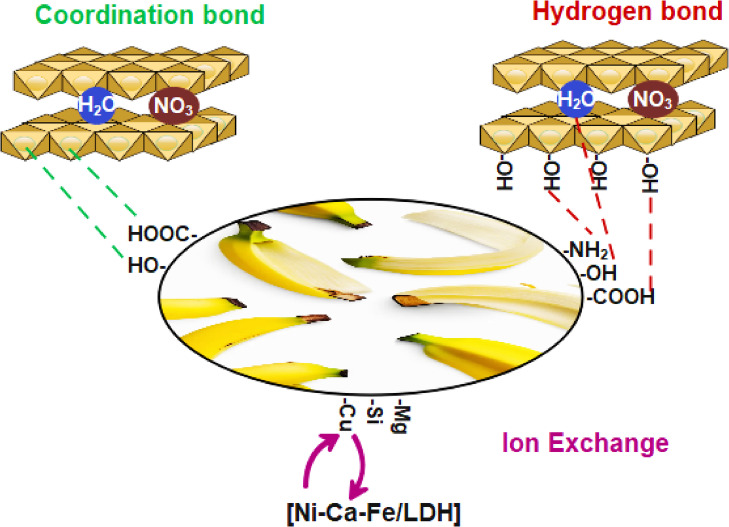
Possible interactions between BP and Ni-Ca-Fe/LDH.

The FTIR spectrum of the BP/LDH composite (Fig. 4C) exhibits noticeable changes and enhanced intensities in several absorption bands compared to those of the pristine components, indicating strong interactions between BP and LDH. The observed shifts of the O–H and C = O bands toward slightly higher wavenumbers, together with increased peak intensities, suggest the formation of coordination bonds between the hydroxyl or carboxyl groups of BP and the metal ions (Ni, Ca, Fe) in the LDH layers. In addition, hydrogen bonding between the hydroxyl groups of BP and those of the LDH structure may contribute to the observed spectral shifts. As illustrated in Scheme 2, ion exchange between the interlayer nitrate ions of LDH and organic species or functional groups from BP may also occur, modifying the LDH vibrational modes and producing further changes in the FTIR spectrum. The FTIR spectra of BP, LDH, and BP/LDH after crystal violet (CV) adsorption are presented in Fig. 4D–F. For BP, the O–H stretching band shifts from 3414 to 3428 cm^−1^ after adsorption, accompanied by a noticeable decrease in intensity, confirming the formation of hydrogen bonds between BP functional groups and CV molecules^42^. In addition, clear shifts in the bands at 2926 and 1424 cm^−1^, which are associated with amino acid groups, further support the involvement of these groups in the adsorption process (Fig. 4D).

For LDH, a significant decrease in the intensity of the O–H stretching band at 3429 cm^−1^ and its shift to 3435 cm^−1^ indicate dipole–dipole hydrogen bonding between the dye molecules and hydroxyl groups in the LDH structure^42^ (Fig. 4E) Moreover, the nitrate-related bands at 1615 and 1379 cm^−1^ exhibit noticeable shifts, suggesting interactions between CV molecules and the interlayer nitrate anions, possibly through coordination involving nitrogen-containing groups. In the case of the BP/LDH composite (Fig. 4F), a general reduction in peak intensities is observed after adsorption, which can be attributed to strong intermolecular interactions between the dye molecules and the composite matrix. These spectral changes confirm the strong adsorption affinity of the BP/LDH composite toward crystal violet dye.

#### XRD

**Fig. 5:**
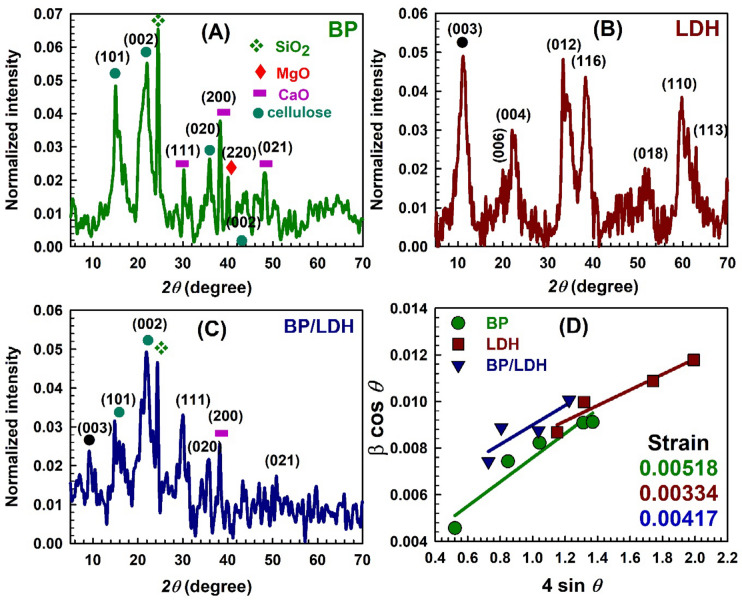
XRD patterns and Williamson − Hall plots of BP, LDH, and BP/LDH.

Figure 5 presents the XRD patterns of the as-prepared BP, LDH, and BP/LDH samples. The X-ray diffractogram of BP (Fig. 5A) exhibits three main diffraction peaks at 2θ = 15.01°, 22.10°, and 35.80°, which correspond to the crystallographic planes (101), (002), and (020) of cellulose, respectively^43,44^. In addition to its organic components, BP contains several inorganic phases such as calcium oxide, magnesium oxide, and silicon dioxide (SiO_2_), which are commonly used as fertilizer materials^44–46^. These findings are consistent with the elemental composition obtained from the EDX analysis shown in Fig. 1.

The XRD pattern of LDH (Fig. 5B) displays characteristic diffraction peaks at 2θ = 11.2°, 20.1°, 22.3°, 33.5°, 38.3°, 52.4°, 59.7°, and 61.2°, which correspond to the (003), (006), (004), (012), (116), (018), (110), and (113) crystallographic planes, respectively. These reflections are in excellent agreement with the standard diffraction patterns of NiFe-LDH (JCPDS card No. 40–0215) and CaFe-LDH (PDF#44–0445) structures^47–49^, confirming the successful synthesis of the LDH phase.

The incorporation of BP into the LDH matrix does not lead to significant changes in the crystalline phase of LDH, as observed in Fig. 5C. However, a noticeable decrease in peak intensity is observed in the composite pattern, which can be attributed to the dilution effect caused by the addition of BP and to the interaction between LDH layers and BP components^50^. Moreover, chemical interactions between LDH and the functional groups of BP may promote the formation of new composite-related phases, particularly the (003) reflection.

Using the Scherrer equation, the average crystallite sizes of BP, LDH, and BP/LDH were calculated to be 31.7, 13.77, and 16.51 nm, respectively. The reduction in crystallite size for the BP/LDH composite indicates that the interaction between BP and LDH leads to the formation of a more complex and finer crystalline structure. To further investigate lattice strain and crystallite size, the Williamson–Hall (W–H) method was employed using the relation^51^:

4 $$\:\:\beta\text{ c}\mathrm{o}\text{s }\theta\:=\:\frac{K\lambda\:}{D}+4\:\epsilon\text{ s}\mathrm{i}\text{n }\theta:$$

The corresponding W–H plots (Fig. 5D) were obtained by linear fitting of β cos θ versus 4 sin θ. The crystallite size (D) was determined from the intercept, while the lattice microstrain (ε) was obtained from the slope. The crystallite sizes calculated from the W–H plots were 57.60, 26.89, and 28.79 nm for BP, LDH, and BP/LDH, respectively, which are in good agreement with the values obtained from Scherrer’s formula. The lattice microstrain values were found to be 0.00518, 0.00334, and 0.00417 for BP, LDH, and BP/LDH, respectively. The higher microstrain value of BP indicates greater lattice distortion, which may arise from structural defects and dislocations commonly present in reactive and layered biomaterials. In contrast, LDH exhibits the lowest microstrain value, reflecting its well-ordered and crystalline layered structure. The intermediate microstrain value of the BP/LDH composite suggests that the LDH matrix helps stabilize the BP structure and reduces its internal lattice stress. Overall, these results confirm the successful formation of the BP/LDH composite with preserved LDH crystallinity, reduced crystallite size, and improved structural stability, which are favorable characteristics for adsorption applications.

### Taguchi model and statistical analysis

**Fig. 6:**
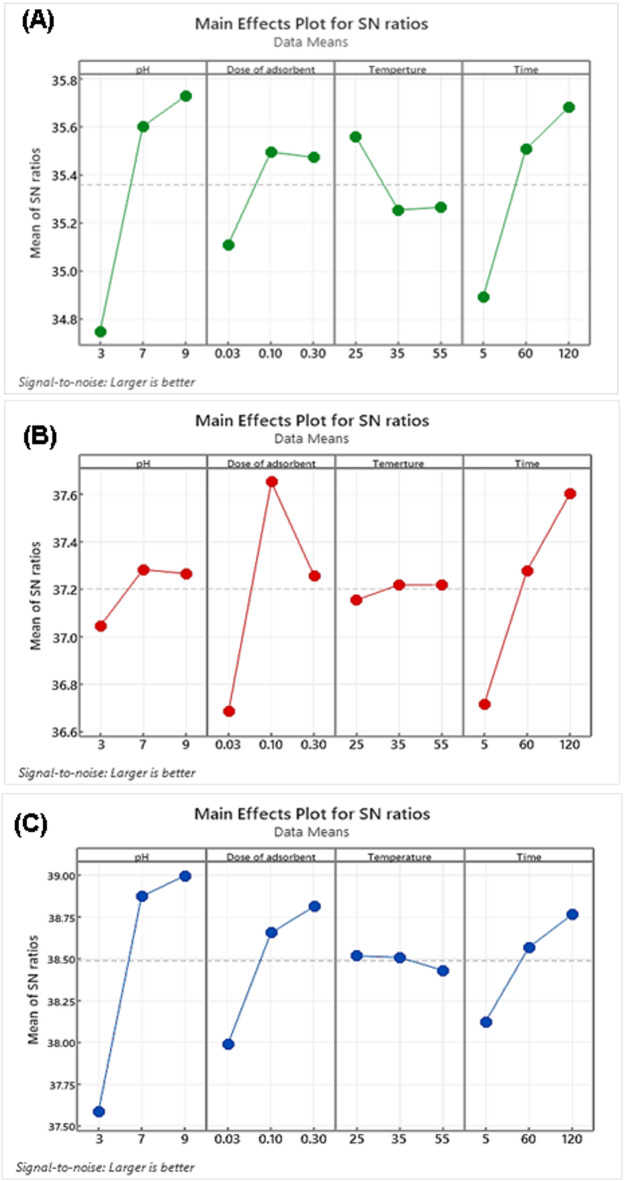
The effect of four factors on the S/N ratio in the removal of CV dye onto BP (**A**), LDH (**B**), and BP/LDH (**C**).

Table 3 presents the experimental results of crystal violet (CV) dye removal obtained using the Taguchi L9 orthogonal array (OA) design, along with the corresponding signal-to-noise (S/N) ratios calculated using Eq. (3). A three-level, four-factor orthogonal matrix was employed to evaluate the effects of contact time, solution pH, adsorbent dosage, and temperature on the dye removal efficiency. A higher S/N ratio indicates better performance and represents the optimal condition for each variable^35^. The S/N ratios of the L9 experimental design matrix are summarized in Table 4. Figure 6 shows the main effects plots for CV removal efficiency as a function of adsorbent dosage, contact time, solution pH, and temperature. The X-axis represents the levels of each input parameter, while the Y-axis indicates the corresponding S/N ratios for dye removal efficiency^52^. These plots are useful for identifying the most influential parameters and their optimal operating levels.

**Table 3:** Experimental design and results for CV dye removal using Taguchi L9 orthogonal array, including observed removal efficiencies and corresponding S/N ratios.

Run no.	Adsorption process variables				Adsorption efficiency (%)		
	pH	Dose (g)	Temperature (°C)	Time (min)	BP	LDH	BP/LDH
1	3	0.03	25	5	51.4778	63.1773	68.9039
2	3	0.10	35	60	59.7290	75.8621	78.2019
3	3	0.30	55	120	56.8288	75.2463	81.9581
4	7	0.03	35	120	60.0369	72.4138	85.8990
5	7	0.10	55	5	57.3891	73.0911	85.4064
6	7	0.30	25	60	63.5467	73.9532	92.4261
7	9	0.03	55	60	59.7906	69.6429	84.4211
8	9	0.10	25	120	65.9482	80.1724	94.1502
9	9	0.30	35	5	58.0049	69.7044	88.9778
Expected response at optimum conditions	65.9483	80.3982	94.1502				

**Table 4:** S/N ratio of the experimental design matrix for L9 orthogonal array.

Run no.	S/*N* ratio		
	BP	LDH	BP/LDH
1	34.2324	36.0112	36.7649
2	34.9289	37.6005	37.8644
3	35.0914	37.5297	38.1403
4	35.5684	37.1964	38.6798
5	35.1766	37.2773	38.6298
6	36.0619	37.3791	39.3159
7	35.5327	36.8575	38.5290
8	36.3841	38.0805	39.4764
9	35.2693	36.8652	38.9856

For BP and BP/LDH, the optimal adsorption conditions were found to be a solution pH of 9, contact time of 120 min, temperature of 25 °C, and an adsorbent dosage of 0.1 g/L. In contrast, the optimal conditions for LDH were a solution pH of 9, contact time of 120 min, temperature of 55 °C, and the same adsorbent dosage of 0.1 g/L. Under these respective optimal conditions, the predicted CV removal efficiencies were 65.94% for BP, 80.40% for LDH, and 94.15% for BP/LDH. The analysis of variance results presented in Table 5 indicates that solution pH has the greatest influence on the adsorption performance of BP and BP/LDH, whereas adsorbent dosage has the most significant effect on LDH. The order of parameter importance was determined as pH > time > dose > temperature for BP, time > dose > pH > temperature for LDH, and pH > dose > time > temperature for BP/LDH.

**Table 5:** Response table for signal to noise ratios.

Adsorbent	Rank			
	pH	Dose (g)	Temperature (°C)	Time (min)
BP	1	3	4	2
LDH	3	2	4	1
BP/LDH	1	2	4	3

### Statistical analysis

Analysis of variance (ANOVA) was performed to evaluate the statistical significance and reliability of the experimental model for CV dye removal. The results, summarized in Table 6, include the sum of squares (SS), mean square (MS), degrees of freedom (DF), F-values, and p-values for each control factor. This analysis was conducted using the S/N ratio and removal efficiency data obtained from the Taguchi L9 experimental design. The F-test was employed to identify the factors that most significantly influence dye adsorption. A higher F-value indicates a stronger impact of the factor on removal efficiency, while a p-value less than 0.05 indicates statistical significance at a 95% confidence level^53^. As shown in Table 6, the F-values for solution pH were the highest for BP and BP/LDH, at 14.37 and 22.12, respectively, indicating that pH is the most influential factor for these adsorbents. In contrast, for LDH, the highest F-value was observed for contact time (3.59), suggesting that contact time has the greatest effect on dye adsorption for this material. The corresponding p-values for pH were 0.019 for BP and 0.009 for BP/LDH, confirming their statistical significance. These findings demonstrate that solution pH is the critical parameter for optimizing CV removal using BP and BP/LDH, whereas contact time plays a more influential role in the adsorption process for LDH.

**Table 6:** Analysis of variance (ANOVA) for adsorption efficiency.

Adsorbent	Parameter	DF	SS	MS	F-Value	*P*-Value
BP	pH	1	72.437	72.437	14.37	0.019
	Dose of adsorbent	1	5.034	5.034	1.00	0.374
	Temperature	1	6.075	6.075	1.20	0.334
	Time	1	41.677	41.677	8.27	0.045
	Error	4	20.168	5.042		
	Total	8	145.391			
LDH	pH	1	5.410	5.4099	0.25	0.646
	Dose of adsorbent	1	10.390	10.3901	0.47	0.530
	Temperature	1	0.058	0.0583	0.00	0.961
	Time	1	79.052	79.0518	3.59	0.131
	Error	4	88.096	22.0240		
	Total	8	183.006			
BP/ LDH	pH	1	301.062	301.062	22.12	0.009
	Dose of adsorbent	1	65.866	65.866	4.84	0.093
	Temperature	1	3.922	3.922	0.29	0.620
	Time	1	50.430	50.430	3.71	0.127
	Error	4	54.434	13.609		
	Total	8	475.714			

### Effect of initial pH and point of zero charge

The solution pH plays a critical role in determining both the surface charge of the adsorbent and the extent of dye adsorption from aqueous media. When the solution pH is below the point of zero charge (pHpzc) of the adsorbent, the surface becomes positively charged, whereas at pH values above pHpzc, the surface carries a negative charge. Under acidic conditions (pH < pHpzc), adsorption efficiency decreases because cationic dye molecules and H^+^ ions compete for the same adsorption sites^43^. In contrast, at alkaline pH (pH > pHpzc), the negatively charged surface enhances electrostatic interactions with the cationic crystal violet (CV) dye. Deprotonation of surface functional groups further increases the number of negatively charged adsorption sites, improving dye uptake.

**Fig. 7:**
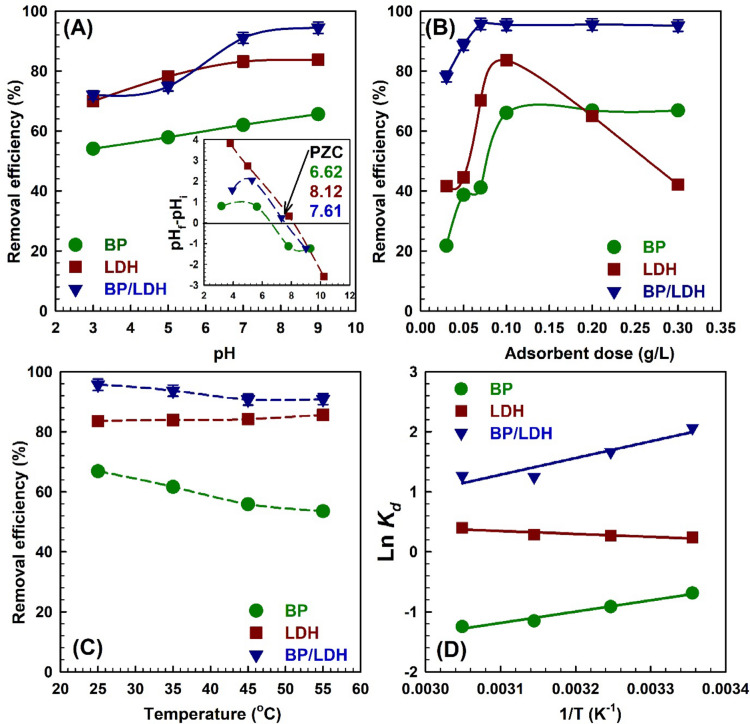
(**A**) The effect of pH solution onto the adsorption of CV dye and including on the figure pH point of zero charge, (**B**) The effect of adsorbent dose, (**C**) Effect of the temperature on the adsorption of CV, and (**D**) van’t Hoff Plot of BP, LDH, and BP/LDH.

Figure 7A shows the determination of the point of zero charge for BP, where ΔpH = 0 occurs at pH 6.62 (pHpzc = 6.62). This value is consistent with previously reported BP pHpzc values of 6.2 and 5.5^54,55^. The LDH and BP/LDH composite exhibit higher pHpzc values of 8.12 and 7.61, respectively, indicating that their surfaces become negatively charged under slightly more alkaline conditions. To investigate the effect of pH on dye removal, batch adsorption experiments were conducted at an initial CV concentration of 20 ppm over a pH range of 3–9. The removal efficiencies increased with pH, reaching maximum values of 65.5, 90.8, and 94.4% for BP, LDH, and BP/LDH, respectively, at pH 9.0 (Fig. 7A). These results demonstrate that pH significantly affects CV adsorption, as the shift of the adsorbent surface from positive to negative charges at pH > pHpzc enhances electrostatic attraction with the positively charged dye molecules. As the pH rises toward 9, the adsorption sites become more favorable for CV binding, resulting in rapid and efficient dye removal until the active sites are fully occupied.

### Effect of adsorbent dose

The effect of adsorbent dosage on CV dye removal was investigated by varying the adsorbent mass from 0.03 to 0.3 g for BP, LDH, and BP/LDH (Fig. 7B). For all three adsorbents, dye removal efficiency increased significantly as the dosage was raised from 0.03 to 0.1 g. This improvement is attributed to the increased number of accessible adsorption sites, which enhances contact between the adsorbent surface and dye molecules. At 0.1 g, the removal efficiencies reached 66.0% for BP and 95.4% for BP/LDH. Further increasing the dosage beyond 0.1 g did not significantly improve adsorption, indicating that equilibrium had been reached and the available active sites were saturated.

In contrast, LDH showed a slightly different trend. Its maximum removal efficiency was also achieved at 0.1 g; however, increasing the dosage further led to a decrease in adsorption efficiency, dropping to 42.08%. This reduction is attributed to bending and aggregation of LDH layers at higher dosages, which reduces the effective surface area and limits accessibility to active sites^56^. Overall, these results highlight that 0.1 g is the optimal adsorbent dosage for efficient CV removal for all materials, while structural factors such as aggregation can negatively affect adsorption for layered LDH.

### Effect of temperature

The effect of temperature on CV adsorption by the three adsorbents is illustrated in Fig. 7C. It was observed that CV adsorption onto BP decreased with increasing solution temperature. This behavior is attributed to the exothermic nature of the adsorption process and the weakening of interactions between dye molecules and adsorbent active sites at elevated temperatures^57^. In contrast, within the investigated temperature range, LDH and the BP/LDH composite maintained relatively stable and high removal efficiencies, with BP/LDH exhibiting the highest performance (95.66%). This thermal stability suggests that adsorption onto LDH-based materials may involve stronger interactions, such as surface complexation or ion exchange, making the process less sensitive to temperature variations. The high adsorption efficiency and thermal stability of BP/LDH further highlight the enhanced surface reactivity and availability of binding sites resulting from the synergistic interaction between BP and LDH phases.

To further understand the adsorption mechanism, thermodynamic parameters including the standard Gibbs free energy change (Δ*G*°), standard enthalpy change (Δ*H*°), and standard entropy change (Δ*S*°) were determined from the adsorption isotherm data. These parameters were calculated using the thermodynamic equilibrium constant (*K*_*d*_), obtained from the best-fitting isotherm model, according to Eqs. (5) and (6)^58^:

5 $$\:\mathrm{ln}{K}_{d}=\frac{{\varDelta\:S}^{o}}{R}-\frac{{\varDelta\:H}^{o}}{RT},$$

6 $$\:{\varDelta\:\mathrm{G}}^{\mathrm{o}}=-RT\mathrm{ln}{K}_{d}.$$

Where *T* is the reaction temperature, *R* is the gas constant (8.314 J/mol K), and *K*_*d*_ = *q*_*e*_/*C*_*e*_ is the thermodynamic equilibrium constant (L/g). *ΔG*^*o*^, *ΔH*^*o*^ and *ΔS*^*o*^ were derived from the slope and intercept of van’t Hoff plot of ln *K*_*d*_ versus 1/*T* as shown in Fig. 7D; Table 7^59^. The positive Δ*H°* value for LDH (4.054 kJ/mol) confirms the endothermic nature of CV adsorption onto LDH, indicating that higher temperatures promote adsorption and enhance adsorption capacity. In contrast, the negative Δ*H°* values for BP and BP/LDH (− 15.629 and − 23.107 kJ/mol, respectively) indicate that CV adsorption onto these materials is an exothermic process.

**Table 7:** Thermodynamic parameters for the adsorption of CV dye by BP, LDH, and BP/LDH.

Adsorbent	Δ*H*^o^ (kJ/mol)	Δ*S*^o^ (J/(mol K))	Δ*G*^0^ (kJ/mol)	*R* ^2^			
				25 °C	35 °C	45 °C	55 °C
BP	-15.629	-58.30	1.700	2.338	3.043	3.396	0.9782
LDH	4.054	15.44	-0.590	-0.686	-0.752	-1.093	0.8170
BP/LDH	-23.107	-60.95	-5.115	-4.259	-3.292	-3.448	0.8952

The entropy change (Δ*S*°) further supports these observations. LDH exhibited a positive Δ*S*° value (15.44 J/mol·K), suggesting increased disorder at the solid–liquid interface, likely due to the displacement of solvent molecules by dye molecules during adsorption^60^. Conversely, BP and BP/LDH showed negative Δ*S*° values (− 58.30 and − 60.95 J/mol·K, respectively), indicating a decrease in randomness, which can be attributed to the orderly attachment of dye molecules onto the adsorbent surfaces.

The Gibbs free energy change (Δ*G*°) values for BP and BP/LDH were negative over the studied temperature range, confirming that CV adsorption onto these materials is spontaneous and thermodynamically favorable. Moreover, the increasingly negative Δ*G*° values with rising temperature indicate enhanced spontaneity at higher temperatures. In contrast, the positive Δ*G*° values obtained for LDH suggest that, although CV adsorption onto LDH is endothermic, the process is not spontaneous under the investigated conditions. This behavior implies a fundamentally different adsorption mechanism compared with BP and BP/LDH, or that temperature alone is insufficient to overcome the associated entropy limitations^11,60^.

### Adsorption isotherms

**Fig. 8:**
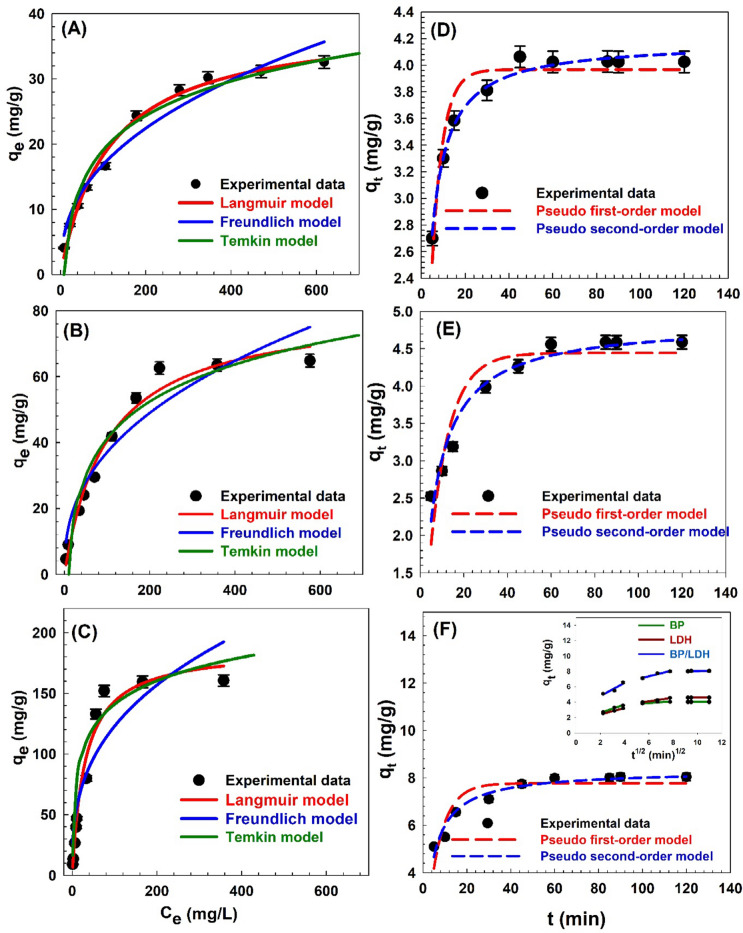
Adsorption isotherms and kinetic of CV on BP (**A**,**D**), LDH (**B**,**E**) and BP/LDH (**C**,**F**) samples, respectively. Inset figure is the intraparticle diffusion model. At pH 9, with a contact time of 120 min, 0.1 g of adsorbent and 25 °C.

Adsorption isotherm models were employed to evaluate the equilibrium adsorption behavior of CV dye onto the adsorbents. The adsorption isotherms of CV onto BP, LDH, and the BP/LDH composite are presented in Fig. 8A–C. Three commonly used isotherm models—Langmuir, Freundlich, and Temkin—were applied to describe and interpret the adsorption process. The Langmuir model assumes monolayer adsorption on a homogeneous surface with a finite number of identical adsorption sites. Once a site is occupied, no further adsorption can occur at that site. The model relates the equilibrium concentration of the adsorbate in solution to the adsorption capacity and the number of available surface sites^4^. In contrast, the Freundlich model is an empirical equation that accounts for multilayer adsorption on heterogeneous surfaces with non-uniform adsorption energies. It describes the relationship between the amount adsorbed and the equilibrium concentration using a power-law expression, reflecting the heterogeneous nature of the adsorption system^11^.

The Temkin isotherm model accounts for indirect adsorbate–adsorbent interactions and provides a more realistic description of chemisorption on heterogeneous surfaces. It assumes that the heat of adsorption decreases linearly with surface coverage due to adsorbate–adsorbent interactions. This model is particularly useful for interpreting adsorption systems in which the binding energy varies with surface loading or coverage^61^. Together, these isotherm models offer mathematical frameworks for evaluating the experimental equilibrium data and elucidating the adsorption mechanism.The corresponding nonlinear forms of the Langmuir, Freundlich, and Temkin equations, presented in Eqs. (7–9), were employed to determine the adsorption parameters and to optimize the performance of the prepared adsorbents^62^.

7 $$\:{q}_{e}={q}_{max}\frac{{{K}_{L}{\mathrm{C}}_{\mathrm{e}}}_{\:}}{1+{K}_{L}{\mathrm{C}}_{\mathrm{e}}}.$$

8 $$\:{q}_{e}={K}_{f}.{{C}_{e}}^{\raisebox{1ex}{$1$}\!\left/\:\!\raisebox{-1ex}{$n$}\right.},$$

9 $$\:\:{q}_{e}=\frac{{RT}}{{b}_{t}}\mathrm{ln}{A}_{t}{C}_{e}.$$

The amount of dye adsorbed at equilibrium is denoted by *q*_*e*_ (mg/g), while the maximum adsorption capacity is represented by *q*_max_ (mg/g). The Langmuir adsorption constant is denoted by *K*_*L*_. The adsorption intensity is represented by *n*, and *K*_*F*_ is the Freundlich adsorption constant. *T* is the absolute temperature (K), *R* is the gas constant (8.3145 J·mol^−1^·K^−1^), and *A*_*t*_ is the equilibrium binding constant (L/g). The Temkin constant related to the heat of adsorption is denoted by *b*_*t*_ (J/mol)^63^.

The equilibrium parameter or separation factor crucial factor is $\:{R}_{L}$, which can be calculated as^59^:

10 $$\:{R}_{L}=\frac{1}{1+{K}_{L}{C}_{o}}.$$

Here, *K*_*L*_ is the Langmuir constant (1/mg) and C_o_ (mg/L) is the greatest dye concentration. The value of *R*_*L*_ indicates weather the isotherm is irreversible (*R*_*L*_ = 0), favorable (0 < 1), linear (*R*_*L*_ = 1), or unfavorable (*R*_*L*_ > 1). The calculated (*R*_*L*_ ​) values for CV adsorption onto BP, LDH, and BP/LDH were found to lie between 0 and 1, indicating favorable adsorption behavior^9^. As presented in Table 8, the regression coefficients (R^2^) for the Langmuir, Freundlich, and Temkin isotherm models are all close to unity, confirming that the experimental adsorption data of CV dye onto BP, LDH, and BP/LDH are well described by these models. Among the three adsorbents, the BP/LDH composite exhibited the highest Langmuir adsorption capacity, demonstrating a stronger affinity for CV dye, particularly at higher concentrations (Fig. 8A–C).

**Table 8:** Parameters calculated by isotherm models for CV adsorption.

Parameters	Adsorbent		
	BP	LDH	BP/LDH
Langmuir Parameters			
R^2^	0.995	0.989	0.985
q_max_ (mg/g)	39.16	81.10	187.40
*K*_*L*_ (L/mg)	0.0088	0.010	0.0326
*R*_*L*_	0.155	0.147	0.160
Freundlich Parameters			
R^2^	0.988	0.950	0.924
n	2.431	2.480	2.844
*K*_*F*_ (mg/g)/(mg/L)^1/n^	2.537	5.780	24.360
Temkin Parameters			
R^2^	0.999	0.999	0.992
*A*_*t*_ (L/g)	0.122	0.126	1.569
*b*_*t*_ (KJ/mol)	0.3253	0.1526	0.0888

The Langmuir isotherm model revealed maximum adsorption capacities (qmax) of 39.16, 81.10, and 187.40 mg/g for BP, LDH, and BP/LDH, respectively. These results clearly indicate a significant enhancement in adsorption capacity upon composite formation. The synergistic interaction between BP and LDH is evidenced by the markedly increased (qmax) value of the BP/LDH composite, which provides a higher density of active adsorption sites. Furthermore, the Langmuir affinity constants (*K*_*L*_) of 0.0088, 0.010, and 0.0326 for BP, LDH, and BP/LDH, respectively, suggest stronger adsorbate–adsorbent interactions in the composite system. The Freundlich model yielded adsorption intensity (*n*) values of 2.431, 2.480, and 2.844 for BP, LDH, and BP/LDH, respectively. Since all *n* values exceed unity, the adsorption process is considered favorable, indicating increased surface heterogeneity. In addition, the substantially higher Freundlich constant (*K*_*f*_) of BP/LDH further confirms its superior adsorption capacity. According to the Temkin model, the heat of adsorption constants (*b*_*t*_) for BP, LDH, and BP/LDH were 0.3253, 0.1526, and 0.0888 kJ/mol, respectively. These low energy values suggest that CV adsorption is predominantly governed by physisorption involving weak van der Waals interactions^64^. Moreover, the BP/LDH composite exhibited a significantly higher Temkin binding constant (*A*_*t*_=1.569 L/g) compared to BP and LDH, indicating stronger binding affinity. Overall, the BP/LDH composite demonstrates the most efficient adsorption performance among the investigated materials, and the isotherm analysis confirms that all three models adequately describe the adsorption behavior of CV dye.

### Adsorption kinetics

Adsorption kinetics provides important insight into the adsorption mechanism and rate-controlling steps by describing the uptake of solute at the solid–solution interface. To analyze the kinetics of CV dye adsorption onto the adsorbents, three kinetic models were applied: the pseudo-first-order, pseudo-second-order, and intra-particle diffusion models. The correlation coefficient (R^2^) was used to evaluate the agreement between the experimental data and the model-predicted values. A relatively high R^2^ value indicates that the model adequately describes the kinetics of CV dye adsorption.

The nonlinear pseudo-first-order (Eq. 11), pseudo-second-order (Eq. (12), and intra-particle diffusion kinetic models (Eq. 13) were applied to the experimental data^65–67^:

11 $$\:{q}_{t}={q}_{e}(1-{e}^{-{K}_{1}t}),$$

12 $$\:{q}_{t}=\frac{{{q}_{e}}^{2}{K}_{2\:}\mathrm{t}}{1+{q}_{e}\:{K}_{2}\mathrm{t}}$$

13 $$\:{{q}_{t}=K}_{ip}\sqrt{t}+{C}_{ip}.$$

Where $\:{q}_{t}$ and $\:{q}_{e}$ (mg/g) are the adsorption capacities at time *t* and at equilibrium, respectively. $\:{K}_{1}$ (min^−1^), *K*_2_ (g/mg.min), and *K*_*ip*_ (mg/g.min^1/2^) are the rate constants of the pseudo-first-order, pseudo-second-order, and intra-particle diffusion models, respectively, while *C*_*ip*_ is the boundary layer thickness constant.

Figure 8D–F present the fitted curves of the pseudo-first-order and pseudo-second-order models, while the intra-particle diffusion model is illustrated in the inset of Fig. 8F. Table 9 summarizes the corresponding kinetic parameters and regression coefficients (R^2^). The kinetic model that best represents the adsorption process was determined based on the goodness of fit using the regression coefficient. The calculated equilibrium adsorption capacities *q*_*e*_ (4.17, 4.87, and 8.32 mg/g) are in close agreement with the experimental values (4.02, 4.59, and 8.04 mg/g for BP, LDH, and BP/LDH, respectively), indicating the reliability of the kinetic models. The excellent fitting is further confirmed by the high correlation coefficients (R^2^ = 0.99, 0.978, and 0.974 for BP, LDH, and BP/LDH, respectively). According to the intra-particle diffusion model, the first steep linear region corresponds to boundary layer diffusion or external mass transfer, where dye molecules rapidly migrate from the solution to the adsorbent surface. This stage is mainly governed by electrostatic interactions and physical adsorption. The second linear region represents intraparticle diffusion, during which dye molecules penetrate into the internal pores of the adsorbent. This stage proceeds more slowly due to pore diffusion resistance and plays a crucial role in controlling the overall adsorption rate. The final plateau region corresponds to the equilibrium stage, where saturation of available active sites leads to a significant reduction in the adsorption rate.

**Table 9:** The fitting results of the adsorption kinetic models and their parameters.

Models	Parameters	BP	LDH	BP/LDH
Pseudo-first-order model	*q*_*e*_ cal (mg/g)	3.960	4.443	7.770
	*K*_*1*_ (min^− 1^)	0.201	0.110	0.154
	R^2^	0.960	0.926	0.900
Pseudo-second-order model	*q*_*e*_ cal (mg/g)	4.17	4.870	8.316
	*K*_*2*_ (g/mg.min)	0.091	0.034	0.061
	R^2^	0.994	0.978	0.974
	*q*_*e*_ exp (mg/g)	4.024	4.587	8.041
intra-particle diffusion model	*K*_*ip*_ (mg/g min^1/2^)	0.545	0.404	0.865
	*C*_*ip*_ (mg/g)	1.507	1.611	4.854
	R^2^	0.983	0.996	0.897

Among the three models, the pseudo-second-order model exhibited the highest R^2^ values, approaching unity, indicating that it best describes the kinetics of CV dye adsorption onto BP, LDH, and BP/LDH. This suggests that the adsorption process is predominantly governed by chemisorption involving valence forces through electron sharing or exchange between the adsorbent surface and dye molecules^67^.

### Adsorption mechanism

**Fig. 9:**
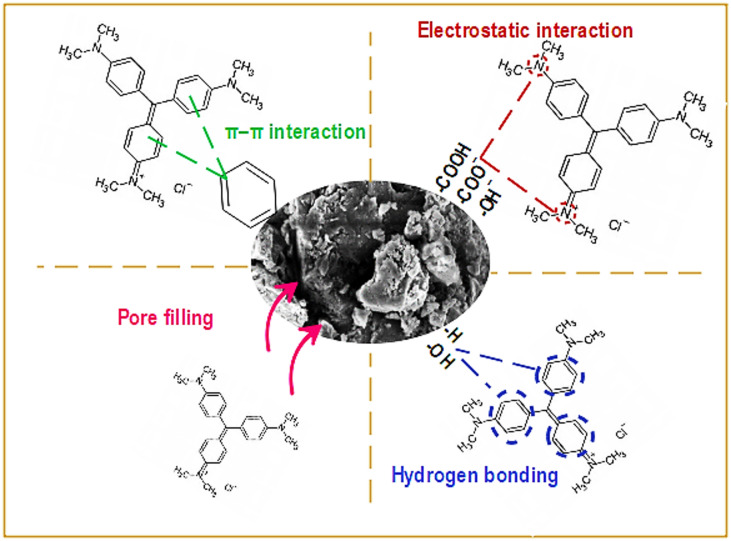
Adsorption mechanism of CV on BP/LDH.

The adsorption mechanism of CV onto the BP/LDH composite is schematically illustrated in Fig. 9, highlighting the contributions of hydrogen bonding, pore filling, electrostatic interactions, and π–π interactions. The aromatic rings of CV interact with the aromatic structures of lignin present in the banana peel matrix through π–π stacking interactions^68–70^, as shown in Fig. 9. In addition, electrostatic attraction occurs between the negatively charged functional groups (COO^−^ and O^−^) on the BP/LDH composite surface and the positively charged (–N^+^) groups of CV. At neutral and alkaline pH, deprotonating of COOH and OH groups increase the negative surface charge, resulting in strong Columbic forces that govern the adsorption process. This is supported by the shifting of nitrate-related bands at 1615 and 1379 cm^−1^ in the FTIR spectrum after adsorption (Fig. 4F), indicating interactions between CV molecules and the interlayer nitrate anions. Furthermore, hydrogen bonding occurs between the nitrogen-containing groups and aromatic hydrogen donors of CV and the hydroxyl groups (OH) on both the LDH layers and banana peel components. The formation of hydrogen bonds is further confirmed by the shift of the O–H stretching band from 3430 to 3414 cm^−1^ after adsorption, accompanied by a noticeable increase in intensity^42^ (Fig. 4F). In addition to these interactions, pore filling contributes significantly to the adsorption mechanism. The porous structure of banana peels combined with the interparticle layers formed by LDH loading provides sufficient space for CV molecules to diffuse into and become physically trapped within the composite’s pores and cavities.

### Effect of adsorbent reusability

Reusability is a crucial factor for the practical application of adsorbents in wastewater treatment. The regeneration performance of BP, LDH, and BP/LDH was evaluated over four consecutive adsorption–desorption cycles under constant experimental conditions. Ethanol was used as the desorbing eluent in multiple regeneration cycles, followed by washing with excess bi-distilled water. Ethanol effectively disrupts the interactions between the adsorbed dye molecules and the active sites of the adsorbents, enabling efficient desorption without damaging the adsorbent structure, while also being an environmentally friendly solvent^71,72^.

**Fig. 10:**
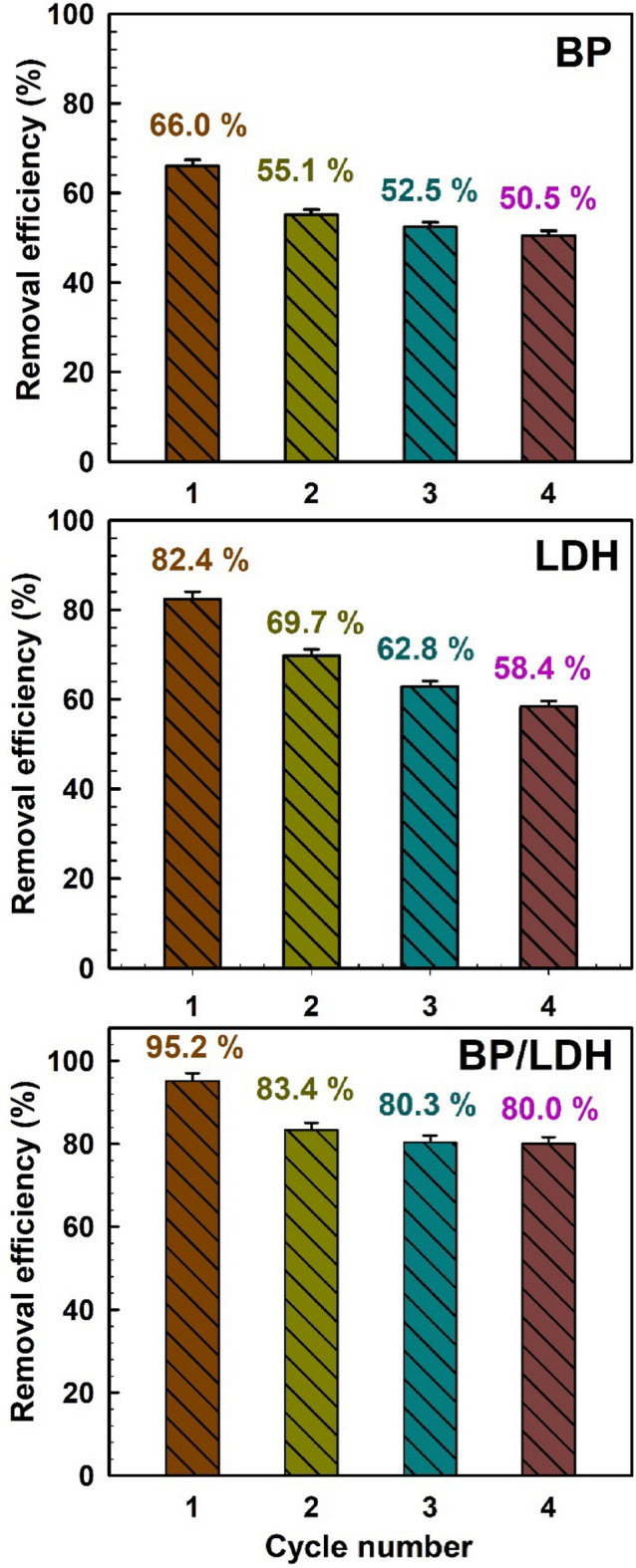
Reusability of BP, LDH, and BP/LDH. At pH 9, with a contact time of 120 min, 0.1 g of adsorbent and 25 °C.

As shown in Fig. 10, the first adsorption cycle exhibited high removal efficiencies of 66.0%, 82.4%, and 95.2% for BP, LDH, and BP/LDH, respectively, owing to the abundance of available active sites. After four cycles, the removal efficiencies decreased to 50.5%, 58.4%, and 80.0% for BP, LDH, and BP/LDH, respectively. This reduction can be attributed to the partial and irreversible occupation of active sites by dye molecules, leading to site saturation and reduced adsorption capacity. Notably, the BP/LDH composite retained relatively high removal efficiency after four cycles, as illustrated in Fig. 10C, indicating superior structural stability and minimal loss of active sites. The slight change in adsorption capacity of BP/LDH after repeated cycles confirms its remarkable regeneration ability and long-term adsorption performance. Furthermore, the adsorption capacity of the synthesized BP/LDH composite toward CV dye was compared with that of several reported adsorbents (Table 10). The BP/LDH composite exhibited a competitive adsorption capacity relative to other bio-based adsorbents, while offering additional advantages in terms of low cost, sustainability, and environmental friendliness^73–78^.

**Table 10:** Comparison of bio-adsorbents with the prepared BP/LDH composite.

Adsorbent	Adsorbate	Maximum Adsorption Capacity (mg/g)	References
Almond shell Marcona (M) Fournat de Brézenaud (FZ) Ferraduel (FD) Ferriages (FG)	Malachite-green dye	41.0 61.0 48.1 118.0	^73^
Charred rice husk (CRH) Xanthated rice husk (XRH)	Crystal violet dye	62.85 90.02	^74^
Carbon sphere (Cs) & titania nanotubes (TNTs)	Crystal violet dye	92.5	^75^
Anchote peel	Methyl orange dye	103.03	^76^
Almond shell (AS)	Methylene blue dye	104.31	^77^
Seed powder of Punica granatum L	Crystal violet dye	434.8	^78^
BP LDH BP/LDH	Crystal violet dye	39.16 81.10 187.40	This work

### Preliminary cost analysis and economic feasibility

A preliminary cost analysis was conducted to evaluate the economic viability of the prepared BP/LDH compound for wastewater treatment applications. Banana peels, used as the main raw material, are an abundant agricultural residue and were obtained at negligible cost. The main expenses are associated with chemical reagents (NaOH and metal nitrate salts) and energy consumption during the activation and drying stages. Based on laboratory-scale preparation, the estimated material cost of the BP/LDH compound is significantly lower than that of conventional activated carbon and many reported nanostructured adsorbents. Furthermore, the high adsorption capacity (187.40 mg/g) and good reusability over multiple cycles substantially reduce the effective cost per treatment cycle. After four reuse cycles, the BP/LDH compound retained approximately 80% of its initial removal efficiency, indicating a low replacement frequency. In addition, the synthesis process is simple, does not require expensive equipment, and can be scaled up using conventional chemical precipitation methods. These factors together suggest that the BP/LDH compound is a cost-effective and economically viable adsorbent for the removal of crystal violet dye from aqueous solutions.

## Conclusion

This study demonstrates that BP, LDH), and their composite (BP/LDH) are effective adsorbents for the removal of CV dye from aqueous solutions, with the BP/LDH composite exhibiting the highest adsorption efficiency. The adsorption kinetics was well described by the pseudo-second-order model, indicating that the adsorption process is mainly controlled by chemisorption. The equilibrium data were satisfactorily fitted by the Langmuir, Freundlich, and Temkin isotherm models, confirming favorable adsorption behavior, as supported by the separation factor (RL) and adsorption intensity (n) values. Optimization using the Taguchi L9 orthogonal array design demonstrated that pH, contact time, adsorbent dosage, and temperature significantly affect the adsorption process. Under the optimized conditions of pH 9, contact time of 120 min, adsorbent dose of 0.1 g, and temperature of 25 °C, the removal efficiencies of CV reached 80.19, 65.94, and 94.15% for LDH, BP, and BP/LDH, respectively. The maximum adsorption capacities were found to be 39.16, 81.10, and 187.40 mg/g for BP, LDH, and BP/LDH, respectively, confirming the superior adsorption performance of the composite material. Thermodynamic analysis revealed that the adsorption of CV onto BP and BP/LDH was spontaneous and exothermic, whereas adsorption onto LDH was endothermic and nonspontaneous. Intraparticle diffusion studies indicated that both boundary layer diffusion and internal pore diffusion contributed to the overall adsorption process through multiple stages. The adsorption mechanism was governed by a combination of physical adsorption, hydrogen bonding, electrostatic interactions, and π–π interactions between CV molecules and the functional groups present on the BP/LDH surface. Regeneration experiments showed that after four adsorption–desorption cycles, the adsorption efficiencies of BP, LDH, and BP/LDH decreased to 58.4, 50.5, and 80.0%, respectively, demonstrating that the BP/LDH composite retains good reusability and structural stability. Overall, the BP/LDH composite derived from agricultural waste represents a promising, low-cost, and environmentally friendly adsorbent for the treatment of dye-contaminated wastewater. Future research should focus on evaluating its performance in real wastewater systems, investigating long-term regeneration stability, optimizing synthesis conditions for large-scale production, and assessing its capability for the simultaneous removal of multiple contaminants.

## Data Availability

The datasets used and/or analysed during the current study are available from the corresponding author on reasonable request.
